# The *TGR5* gene is expressed in human subcutaneous adipose tissue and is associated with obesity, weight loss and resting metabolic rate

**DOI:** 10.1016/j.bbrc.2013.03.031

**Published:** 2013-04-19

**Authors:** Per-Arne Svensson, Maja Olsson, Johanna C Andersson-Assarsson, Magdalena Taube, Maria J. Pereira, Philippe Froguel, Peter Jacobson

**Affiliations:** a Department of Molecular and Clinical Medicine and Center for Cardiovascular and Metabolic Research, Institute of Medicine, The Sahlgrenska Academy, University of Gothenburg, Gothenburg, Sweden; b Sahlgrenska Cancer Center, Department of Molecular and Clinical Medicine, Institute of Medicine, The Sahlgrenska Academy, University of Gothenburg, Gothenburg, Sweden; c The Lundberg Laboratory for Diabetes Research, Department of Molecular and Clinical Medicine, Institute of Medicine, The Sahlgrenska Academy, University of Gothenburg, Gothenburg, Sweden; d Center for Neuroscience and Cell Biology, University of Coimbra, Portugal; e Department of Genomics of Common Disease, School of Public Health, Imperial College London, Hammersmith Hospital, London W12 0NN, UK; f Centre National de la Recherche Scientifique-8090, Institute of Biology, Pasteur Institute, 59019 Lille, France

**Keywords:** *TGR5*, Human adipose tissue, Gene expression, Obesity, Resting metabolic rate

## Abstract

Bile acids have emerged as a new class of signaling molecules that play a role in metabolism. Studies in mice have shown that the bile acid receptor *TGR5* mediates several of these effects but the metabolic function of *TGR5* in humans is less well established. Here we show that human adipose tissue *TGR5* expression is positively correlated to obesity and reduced during diet-induced weight loss. Adipose tissue *TGR5* expression was also positively correlated to resting metabolic rate. Our study indicates that human adipose tissue contributes to the *TGR5* mediated metabolic effects of bile acids and plays a role in energy expenditure.

## Highlights

• Human adipose tissue (AT) expresses the bile acid receptor *TGR5*.
• Human AT *TGR5* expression is linked to obesity.
• Resting metabolic rate and AT *TGR5* expression is positively correlated.
• *TGR5* expression is not higher in brown compared to white human AT.

## 1 Introduction

Bile acids are synthesized in the liver and play a central role in dietary lipid emulsification in the intestine. The majority of the secreted bile acids is taken up by the distal parts of the small intestine and return to the liver and the gall bladder via the enterohepatic circulation. Beyond their well established function in dietary lipid emulsification, bile acids are signaling molecules with metabolic effects mediated by specific receptors. The farnesoid X receptor (FXR) is the most studied receptor for bile acids. FXR is a nuclear receptor that regulates bile acid synthesis as well as lipid and glucose metabolism [1]. The G protein-coupled receptor *TGR5* (also denoted G protein-coupled bile acid receptor 1 or GBPAR1) has also been found to be a functional bile acid receptor [2]. In 2006, Watanabe et al. showed that bile acid administration augmented energy expenditure in mice [3], and that these effects were mediated via the *TGR5* and its effects on the thyroid hormone activating enzyme iodothyronine deiodinase type II (DIO2) in brown adipose tissue (BAT). In addition, they showed that bile acid treatment of human skeletal myocytes increased both DIO2 activity and oxygen consumption, indicating that similar mechanisms are relevant also in humans. More recently, *TGR5* activation has been shown to induce glucagon-like peptide-1 (GLP-1) release in mice, indicating a direct role for *TGR5* in glucose homeostasis [4].

Despite these advances in understanding of *TGR5*, much less in known about *TGR5* function in humans and its expression in other metabolically relevant organs such as adipose tissue. The aim of this study was therefore to investigate the potential role of adipose tissue *TGR5* in human metabolism.

## 2 Materials and methods

The regional ethics committee in Gothenburg approved these studies.

### 2.1 Sib Pair study

The swedish obese subjects (SOS) Sib Pair study consists of 154 nuclear families with BMI discordant sibling pairs (BMI difference ⩾10 kg/m^2^), resulting in a study population consisting of 732 subjects [5]. The subjects were extensively phenotyped [5], including anthropometric measurements and determination of resting metabolic rate (RMR) in a ventilated hood [6]. Subcutaneous adipose tissue needle biopsies were obtained and used for gene expression analysis. For the current study, complete data from 353 siblings and 86 parents were available for the analysis.

### 2.2 Very low calorie diet (VLCD) study

The very low calorie diet (VLCD) study was performed to investigate gene expression changes in adipose tissue of obese subjects during weight loss induced by caloric restriction. Twenty-eight obese subjects (20 women and 8 men, age 39.7 ± 12.7 years, BMI 36.3 ± 3.7 kg/m^2^) were treated with VLCD (450 kcal/day) for 12 weeks [7,8]. Subcutaneous adipose tissue needle biopsies were obtained at the start of the VLCD treatment (day 0) and three times during the VLCD treatment (weeks 2, 6, and 12). After 12 weeks of VLCD treatment, the mean weight loss was 19%.

### 2.3 Perithyroid and perirenal adipose tissue studies

Perthyroid adipose tissue surgical samples were obtained from 24 patients undergoing surgery in the thyroid region for malignancies or endocrine disorders. Clinical characteristics of the patients have been described previously [9]. Perithyroid adipose tissue biopsies containing BAT was identified by expression analysis of uncoupling protein 1 (*UCP1*) and histological analysis. Samples with high *UCP1* expression was classified as BAT positive (BAT+, *n* = 9). Paired subcutaneous white adipose tissue biopsies from the surgical incision area were also obtained from the same nine patients (2 men and 7 women, age 47 ± 21 years, BMI 23 ± 2 kg/m^2^).

Biopsies of perirenal adipose tissue were obtained from 55 healthy kidney donors. The perirenal adipose tissue samples were screened for *UCP1* expression and samples with high *UCP1* expression (samples from 4 men and 6 women) were classified as BAT positive (BAT+) samples. These samples constituted the BAT+ group. A control group (*n* = 10) with low perirenal adipose tissue *UCP1* expression (classified as BAT− samples) was matched to the BAT+ group based on sex, age and BMI. The subjects in the BAT+ group had an average age of 42 ± 14 years and a BMI of 26 ± 2. The subjects in the BAT− group had an average age of 44 ± 9 years and a BMI of 26 ± 3.

### 2.4 Gene expression analysis

Total RNA was isolated from adipose tissue using the RNeasy lipid tissue midi kit (Qiagen, Chatsworth, CA) or the phenol–chloroform extraction method of Chomczynski and Sacchi [10].

Gene expression in adipose tissue from the Sib Pair study and in the perithyroid adipose tissue was analyzed study using Human Genome U133 plus 2.0 arrays (Affymetrix, Santa Clara, CA). Gene expression in the perirenal adipose tissue samples were analyzed by (Affymetrix Gene 1.0 ST arrays at the Uppsala Array Platform, Uppsala, Sweden). All arrays were analyzed according to the manufacturer’s instructions. Expression data were analyzed using the RMA algorithm (Affymetrix). *TGR5* expression was assessed using probe sets 1552501_a_at and 8048249 for Human Genome U133 plus 2.0 and Expression assay Gene 1.0 ST arrays, respectively.

Adipose tissue total RNA from the VLCD study was reversed transcribed using the High Capacity cDNA RT kit (Life Technologies, Paisley, UK) according to the manufacturer’s protocol. Reagents for real-time PCR analysis of *TGR5* (Hs00544894_m1) and low-density lipoprotein (LDL) receptor-related protein 10 (LPR10) (Hs00204094_m1) were purchased from Life Technologies and used according to the manufacturer’s instructions. cDNA was used for real-time PCR in the Applied Biosystems PRISM 7900HT Sequence Detection System (Life Technologies) using default cycle parameters. A standard curve was plotted for each primer-probe set with a serial dilution of cDNA synthesized from pooled RNA. All samples and standards were analyzed in triplicate and *LRP10* gene expression was used as a reference gene [11].

### 2.5 Gene ontology (GO) enrichment analysis

GO enrichment analysis was performed using the DAVID web–accessible program [12,13] (http://david.abcc.ncifcrf.gov). Transcripts significantly correlated with *TGR5* expression were identified in the Sib Pair study using Spearman correlation. Accounting for multiple testing, a threshold of *p* < 9.1 × 10^−7^ was used as a cut off for declaring statistical significance (Bonferroni correction). This yielded a total of 1755 negative and 1767 positivity correlated transcripts which were included separately in the GO enrichment analysis. The analysis was limited to the GOTERM_BP_FAT, GEOTERM_CC_FAT and GEOTERM_MF_FAT options and Human Genome U133 Plus 2.0 was used as a background.

### 2.6 Statistical analysis

Statistical analyses were performed using the SAS software package (v. 9.1.3, SAS Institute Inc., Cary, NC) or and PASW Statistics (Chicago, IL, USA). Quantitative data were transformed towards normal distribution using Box–Cox power transformations. Outliers beyond three standard deviations from the trait mean were excluded. Correlations between clinical traits and *TGR5* expression were analyzed using the MIXED procedure in SAS. In the linear mixed models, we used a “sandwich estimator” of the covariance matrix to adjust for non-independence among family members. This asymptotically yields the same parameter as ordinary least squares or regression methods while standard errors and, consequently, hypothesis tests are adjusted for the family relatedness. Comparisons in gene expression between BAT and WAT were performed using Student’s *t*-test (paired or unpaired as appropriate). Gene expression during weight loss was analyzed using a one-way repeated measures analysis of variance (ANOVA) with Greenhouse–Geisser correction. Comparisons between time points were assessed by Bonferroni corrected post-hoc tests.

## 3 Results

### 3.1 TGR5 adipose tissue expression

**Fig. 1:**
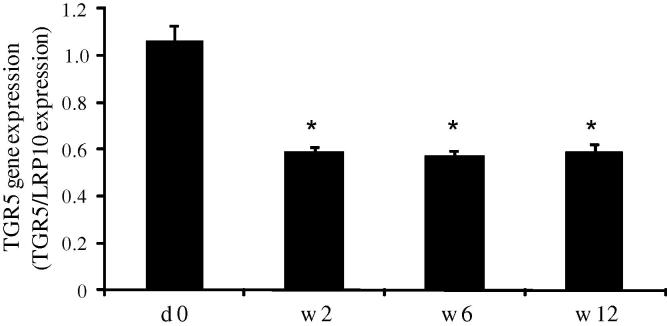
Adipose tissue *TGR5* expression during weight loss. Twenty-eight obese patients (20 women and 8 men) were treated with a very low caloric diet for 12 weeks. Subcutaneous adipose tissue biopsies were obtained by needle aspiration before the diet (day 0, d0) and after 2, 6 and 12 weeks (w) of diet. *TGR5* gene expression was analyzed by real-time PCR and normalized to the reference gene *LRP10*. Data is presented as mean ± SEM. ^∗∗^*p* < 0.0005 compared with d0.

Initially, we investigated the link between *TGR5* adipose tissue expression and obesity. Among the Sib Pair families, *TGR5* expression was positively correlated with BMI (*r* = 0.40, *p* < 0.0001), independent of age, sex, family membership and generation. In line with this finding, obese subjects treated with VLCD displayed a drastic reduction of adipose tissue *TGR5* expression determined by a repeated measures ANOVA (*F*(1.4, 38) = 38, *p* < 0.0005; Fig. 1). Compared to day 0, adipose tissue *TGR5* expression was approximately half as low during the entire dieting period and post-hoc tests using the Bonferroni correction showed that *TGR5* levels were reduced compared with day 0 at all subsequent time points measured (weeks 2, 6 and 12, *p* < 0.0005 for each test; Fig. 1).

### 3.2 Gene ontology enrichment analysis

To gain insights into functions in adipose tissue that may be related to *TGR5* expression, Gene ontology enrichment analysis was performed using transcripts positively or negatively correlated to *TGR5* expression in subcutaneous adipose tissue in the Sib Pair offspring study. Transcripts negative correlated to *TGR5* expression displayed highly significant (adjusted *p*-values down to 3 × 10^−20^) enrichment of several GO terms relating to mitochondria and ribosome (Table 1). Enrichment analysis of transcripts positively correlated to *TGR5* expression resulted in identification of GO terms primarily related to endoplasmatic reticulum/Golgi and cytoskeleton function but with less striking *p*-values (Table 1).

### 3.3 TGR5 adipose tissue expression and energy expenditure

Since mitochondria play an important role in energy expenditure and nutrient combustion, we next investigated the relation between adipose tissue *TGR5* expression and resting metabolic rate (RMR) in the Sib Pair study. Unexpectedly, given the negative correlation to mitochondrial genes, *TGR5* expression was positively correlated with RMR (*r* = 0.25, *p* < 0.0001). In a model including age, polynomials of age, sex, lean body mass, generation and family membership, the statistical significance of this correlation persisted.

**Fig. 2:**
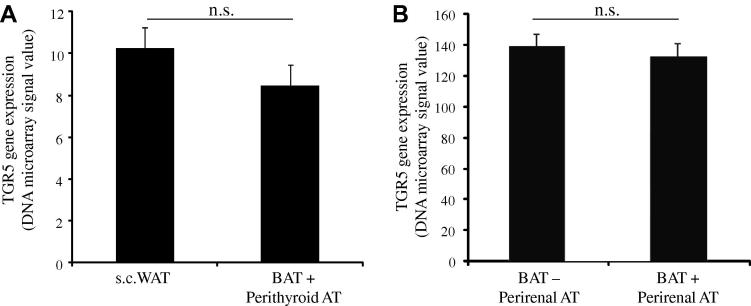
*TGR5* gene expression in human brown and white adipose tissue. (A) *TGR5* expression in BAT containing (BAT+) perithyroid adipose tissue and paired subcutaneous white adipose tissue (s.c. WAT, *n* = 9). (B) *TGR5* expression in perirenal adipose tissue containing BAT (BAT+, *n* = 10) or only containing white adipose tissue (BAT−, *n* = 10). Data is presented as mean microarray signal ± SEM. n.s. = not significant. *Note*: the different signal values are due to different microarray types.

Brown adipocytes have, in comparison to white adipocytes, substantially more mitochondria. In mice *TGR5* has been shown to be expressed in brown adipose tissue (BAT) and treatment of brown adipocytes with bile acids has been shown to increase oxygen expenditure via *TGR5*-mediarted activation of *DIO2* in BAT [3]. The expression of *TGR5* was therefore investigated in two sets of human adipose tissue samples (the perithyriod and the perirenal adipose tissue studies) containing islets of BAT (BAT+) and compared to expression in white adipose tissue. However, no significant difference in *TGR5* expression was observed between the BAT containing samples and white adipose tissue (Fig. 2A and B).

## 4 Discussion

In this study, we have characterized the gene expression of *TGR5* in human adipose tissue. Our main findings are that *TGR5* adipose tissue expression is reduced during weight loss, and that it is positively correlated with obesity as well as with resting metabolic rate.

Our results clearly show that *TGR5* expression in adipose tissue is regulated in a manner that supports a functional metabolic role for *TGR5* in adipose tissue. However, our results are not easily reconciled with the results from the studies of mice treated with bile acids [3]. Whereas mice treated with bile acids lose weight, high human adipose tissue *TGR5* expression is associated with obesity. In addition, the mice studies also show that the mitochondria rich BAT is a major target organ for the effects of bile acids whereas we show a negative correlation of *TGR5* to the expression of mitochondrial genes in white adipose tissue. In the light of this, it is not unthinkable that the up regulation of *TGR5* in human adipose tissue can be viewed as compensatory mechanism to prevent adipose tissue growth. However, one has to consider that regulation studies in one single organ may not capture the more complex regulation of the entire body and that receptor gene or protein expression cannot directly be translated to the amount of receptor signaling.

Expression analysis in human brown fat depots is challenging because, in contrast to mouse, human BAT represents a much smaller fraction of adipose tissue and is usually not located in distinct depots but rather as islets within the white adipose tissue [9,14]. Only the expression of a very limited number of genes has been investigated in human BAT [9,15,16]. Our analysis of *TGR5* expression in human BAT-containing adipose tissue samples provide no evidence that *TGR5* is expressed at higher levels in human BAT compared to WAT. This together with the limited amount of BAT in humans speaks against that the observed association between *TGR5* adipose tissue expression and RMR is related to human BAT. A more likely scenario is that *TGR5* expression in adipose tissue is co regulated with the *TGR5* expression in other metabolically more active organs such as skeletal muscle or that it is related to adipose tissue thermogenesis not involving BAT. The correlation between adipose tissue *TGR5* expression and RMR is well in line with the down-regulation of *TGR5* during the diet intervention, a situation where RMR is reduced [17]. Interestingly, the drastic reduction of *TGR5* expression seen already after 2 weeks preceded the maximal weight loss which occurred at week 12 [8]. This indicates that *TGR5* expression in adipose tissue is regulated by energy intake or metabolic rate rather than the level of obesity. One study has shown that human plasma levels of bile acids are not associated with energy metabolism [18], indicating that if these systems play an important role in human energy expenditure future research should probably be directed at the receptor level.

Obesity represents an independent cardiovascular risk factor [19]. In addition to its metabolic role, *TGR5* has recently been directly implicated in the development of atherosclerosis. Activation of *TGR5* in mice was associated with attenuation of atherosclerosis by means of decreased pro-inflammatory cytokine production and reduced oxidized LDL uptake in macrophages [20]. These findings are of particular interest to our study since adipose tissue is a major source of circulating pro-inflammatory cytokines. However, the relative contribution of the adipose tissue *TGR5* expression by adipocytes and macrophages is currently unknown.

In conclusion, we have shown that adipose tissue expression of human *TGR5* is reduced during weight loss, and positively correlates to obesity as well as to resting metabolic rate, indicating a functional metabolic role for *TGR5* in adipose tissue. However, more studies are needed to specifically define this role.

## Figures and Tables

**Table 1:** Gene ontology enrichment analysis of transcripts correlated to *TGR5* expression in adipose tissue in the Sib Pair study.

GO term	Category	Count^a^	Adj *p*-value^b^
*Transcripts negatively correlated to TGR5 expression*			
Mitochondrion	CC	160	3.0E−20
Mitochondrial part	CC	100	8.2E−16
Intracellular organelle lumen	CC	203	4.4E−13
Organelle lumen	CC	205	7.5E−13
Membrane-enclosed lumen	CC	208	9.0E−13
Translation	BP	67	1.6E−11
Ribosome	CC	46	7.1E−11
Mitochondrial envelope	CC	68	6.1E−10
Organelle inner membrane	CC	58	6.3E−10
Mitochondrial membrane	CC	64	1.9E−09
Mitochondrial lumen	CC	46	2.2E−09
Mitochondrial matrix	CC	46	2.2E−09
Ribosomal subunit	CC	32	3.5E−09
Ribonucleoprotein complex	CC	75	4.8E−09
Mitochondrial inner membrane	CC	53	6.1E−09

*Transcripts positively correlated to TGR5 expression*			
Endoplasmic reticulum	CC	135	5.7E−08
Actin binding	MF	54	0.000023
Cytoskeletal protein binding	MF	74	0.000031
GOLGI apparatus	CC	115	0.000033
Lysosome	CC	41	0.00006
Lytic vacuole	CC	41	0.00006
Endoplasmic reticulum part	CC	54	0.00067
Endoplasmic reticulum lumen	CC	21	0.00078
Vacuole	CC	42	0.0014
Actin cytoskeleton organization	BP	39	0.0019
Cortical cytoskeleton	CC	15	0.0019
Actin filament-based process	BP	41	0.0023
Cell cortex	CC	28	0.0023
Proteinaceous extracellular matrix	CC	48	0.0023
Extracellular matrix part	CC	24	0.0027

CC = cellular component; BP = biological process; MF = molecular function.

a Number of correlated transcripts included in the specific GO term.

b The table displays the top 15 enriched GO terms based on the adjusted *p*-value (Benjamini).
